# Lessons Learned from the Pilot Phase of a Population-Wide Genomic Screening Program: Building the Base to Reach a Diverse Cohort of 100,000 Participants

**DOI:** 10.3390/jpm12081228

**Published:** 2022-07-27

**Authors:** Caitlin G. Allen, Leslie Lenert, Kelly Hunt, Amy Jackson, Elissa Levin, Catherine Clinton, John T. Clark, Kelli Garrison, Sam Gallegos, Karen Wager, Wenjun He, Katherine Sterba, Paula S. Ramos, Cathy Melvin, Marvella Ford, Kenneth Catchpole, Lori McMahon, Daniel P. Judge

**Affiliations:** 1 Department of Public Health Sciences, Medical University of South Carolina, Charleston, SC 29425, USA; huntke@musc.edu (K.H.); jacksamy@musc.edu (A.J.); clarkjt@musc.edu (J.T.C.); garrisok@musc.edu (K.G.); gallegsa@musc.edu (S.G.); hewwe@musc.edu (W.H.); sterba@musc.edu (K.S.); melvinc@musc.edu (C.M.); 2 Biomedical Informatics Center, Medical University of South Carolina, Charleston, SC 29425, USA; lenert@musc.edu; 3 Clinical & Policy, Helix, San Mateo, CA 94401, USA; elissa.levin@helix.com (E.L.); catherine.clinton@helix.com (C.C.); 4 Department of Healthcare Leadership and Management, College of Health Professions, Medical University of South Carolina, Charleston, SC 29425, USA; wagerka@musc.edu; 5 Department of Medicine, Department of Public Health Sciences, Medical University of South Carolina, Charleston, SC 29425, USA; ramosp@musc.edu; 6 Hollings Cancer Center, Medical University of South Carolina, Charleston, SC 29425, USA; fordmar@musc.edu; 7 Anesthesia & Perioperative Medicine, Medical University of South Carolina, Charleston, SC 29425, USA; catchpol@musc.edu; 8 Office of Vice President for Research, Department of Neuroscience, Medical University of South Carolina, Charleston, SC 29425, USA; mcmahonl@musc.edu; 9 Division of Cardiology, Medical University of South Carolina, Charleston, SC 29425, USA; judged@musc.edu

**Keywords:** precision public health, genomic screening, population screening, implementation science

## Abstract

*Background and Objectives*: Genomic information is increasingly relevant for disease prevention and risk management at the individual and population levels. Screening healthy adults for Tier 1 conditions of hereditary breast and ovarian cancer, Lynch syndrome, and familial hypercholesterolemia using a population-based approach can help identify the 1–2% of the US population at increased risk of developing diseases associated with these conditions and tailor prevention strategies. Our objective is to report findings from an implementation science study that evaluates multi-level facilitators and barriers to implementation of the *In Our DNA SC* population-wide genomic screening initiative. *Methods:* We established an IMPACTeam (IMPlementAtion sCience for *In Our DNA SC* Team) to evaluate the pilot phase using principles of implementation science. We used a parallel convergent mixed methods approach to assess the Reach, Implementation, and Effectiveness outcomes from the RE-AIM implementation science framework during the pilot phase of *In Our DNA SC*. Quantitative assessment included the examination of frequencies and response rates across demographic categories using chi-square tests. Qualitative data were audio-recorded and transcribed, with codes developed by the study team based on the semi-structured interview guide. *Results:* The pilot phase (8 November 2021, to 7 March 2022) included recruitment from ten clinics throughout South Carolina. Reach indicators included enrollment rate and representativeness. A total of 23,269 potential participants were contacted via Epic’s MyChart patient portal with 1976 (8.49%) enrolled. Black individuals were the least likely to view the program invitation (28.9%) and take study-related action. As a result, there were significantly higher enrollment rates among White (10.5%) participants than Asian (8.71%) and Black (3.46%) individuals (*p* < 0.0001). Common concerns limiting reach and participation included privacy and security of results and the impact participation would have on health or life insurance. Facilitators included family or personal history of a Tier 1 condition, prior involvement in genetic testing, self-interest, and altruism. Assessment of implementation (i.e., adherence to protocols/fidelity to protocols) included sample collection rate (*n* = 1104, 55.9%) and proportion of samples needing recollection (*n* = 19, 1.7%). There were no significant differences in sample collection based on demographic characteristics. Implementation facilitators included efficient collection processes and enthusiastic clinical staff. Finally, we assessed the effectiveness of the program, finding low dropout rates (*n* = 7, 0.35%), the identification of eight individuals with Tier 1 conditions (0.72% positive), and high rates of follow-up genetic counseling (87.5% completion). *Conclusion:* Overall, Asian and Black individuals were less engaged, with few taking any study-related actions. Strategies to identify barriers and promoters for the engagement of diverse populations are needed to support participation. Once enrolled, individuals had high rates of completing the study and follow-up engagement with genetic counselors. Findings from the pilot phase of *In Our DNA SC* offer opportunities for improvement as we expand the program and can provide guidance to organizations seeking to begin efforts to integrate population-wide genomic screening.

## 1. Introduction

Genetic information is increasingly relevant for disease prevention and risk management at the individual and population levels [1,2]. Rapidly decreasing sequencing costs and increased throughput ability have paved the path for population-level genetic and genomic testing to support precision medicine and population health [3,4]. In 2018, the Genomics and Population Health Action Collaborative of the National Academies of Science, Engineering, and Mathematics developed a roadmap for the implementation of population-wide genomic screening programs for Centers for Disease Control and Prevention’s (CDC) Tier 1 conditions [5]. Screening for Tier 1 conditions of hereditary breast and ovarian cancer, Lynch syndrome, and familial hypercholesterolemia among healthy adults with or without family history can identify the 1–2% of the U.S. population at increased risk of developing diseases associated with these conditions. Once an individual is identified with increased risk, established interventions are available to reduce overall morbidity and mortality.

Despite the accessibility of genetic information and growth in population-based screening, challenges exist to scaling up these approaches, including engaging large multidisciplinary teams of researchers and clinicians, ensuring public understanding of genetic information, equitable access and participation of diverse populations in genetic screening, and sustainability of population-based genetic screening programs [6,7]. Synergistic efforts to optimally use genomic information to inform clinical care and improve population health requires the use of implementation science to assess engagement with learning health systems, define and monitor project outcomes, and refine and evaluate processes for improvement.

In 2021, the Medical University of South Carolina (MUSC) partnered with Helix, a leading population genomics company, to offer population-level genomic testing. This partnership, called *In Our DNA SC*, is designed to provide genetic testing for up to 100,000 participants for CDC Tier 1 conditions. *In Our DNA SC* uses a multi-phased implementation approach, including a pilot phase of program implementation at 10 clinical sites, institutional expansion across clinical sites affiliated with MUSC, and community expansion to people not previously affiliated with MUSC. As part of the program, we established an IMPACTeam (IMPlementAtion sCience for *In Our DNA SC* Team) to create a strategy evaluation of the program using principles of implementation science [8]. The purpose of this article is to report the reach, implementation, and effectiveness outcomes from the pilot phase of the program, the lessons learned, and next steps to facilitate population-wide screening, both at MUSC and elsewhere.

## 2. Methods

### 2.1. Setting and Sample

We describe findings from the pilot phase of the *In Our DNA SC* program, which took place in 10 MUSC-affiliated outpatient clinics over a 3-month period (8 November 2021 through 7 March 2022, inclusive) with the goal of enrolling 2694 participants. The study team selected clinical sites for implementation based on the proportion of the patient population with active patient portal (Epic MyChart) accounts, geographic distribution, and patient volume. Eligibility to participate included: being an adult (18+), ability to speak English, does not have primary residency in New York State, and having a clinical visit at a participating clinic within the next 7 days. Individuals received a message through the patient portal alerting them of their eligibility to participate in the study. If individuals did not respond to the initial message, a follow-up message was sent through MyChart three days before their visit. If an individual expressed interest through their MyChart account, a study team member then sent a follow-up message through the patient portal with detailed instructions about enrollment and initiated a phone call. Once consented, a standing order was automatically generated for sample collection during the upcoming routine appointment. Individuals were provided with instructions about the process for completing sample collection at their appointment. Trained clinical staff provided the specimen collection kit at the patient’s appointment and returned the completed kit to the Helix laboratory for processing. Participants and their providers received their results via their patient portal approximately 8–12 weeks after initial collection. Research staff attempted to contact individuals three times if they tested positive for one of the hereditary conditions prior to releasing the results to patient records. Those who tested positive were offered free genetic counseling with genetic counselors at MUSC.

### 2.2. Design and Data Collection

We used a parallel convergent mixed methods design to assess the reach, effectiveness, and implementation outcomes during the pilot phase of the program from the RE-AIM framework [9]. Reach is defined as the number and representativeness of participants compared to the intended audience. We operationalized reach as the total number of participants and how well those individuals represented those invited [8]. We also considered qualitative aspects of reach to better understand reasons for enrollment or non-enrollment. Effectiveness is defined as the degree to which the intervention changes a health outcome. Our primary effectiveness outcome is based on the number of individuals who complete the program (i.e., results are returned) and the proportion of participants who are identified with a pathogenic variant for CDC Tier 1 conditions and receive genetic counseling. Implementation focuses on how well the intervention or program is delivered. We operationalized this at the setting level and individual level, with the primary focus of this analysis at the individual level. Specifically, we assessed the characteristics of those who had their sample collected compared to those who did not.

To monitor participation in the program, we developed a SQL database that extracted information from the electronic health record to track patients who received recruitment messages and whether the patients declined, were non-responsive, expressed interest, or enrolled in the project. The database captured information on samples collected, sample re-collection (if the original sample was not sufficient), whether samples were sent to Helix, whether results were returned to the participant, the number of positive individuals who complete genetic counseling, and the number who scheduled additional screening. This database also includes information about participant demographics available from the electronic health record, including gender, race, ethnicity, age, and area of residence.

In addition to the quantitative data gathered, we completed qualitative interviews to further probe areas of drop-off, discrepancies in the anticipated and actual numbers of individuals, or differences in sociodemographics. Interviewees included individuals who did not enroll in *In Our DNA SC*, either because they declined or reviewed the invitation to participate and took no action, and people who participated. Interviews were conducted via MS Teams or phone using a semi-structured interview guide tailored to an individual’s status regarding their participation experience with the program (declined, consented, sample collected). Additional qualitative data were captured using research coordinator tracking logs of questions and calls made during the roll-out of the program. Details about the types of questions and whether follow-up was needed were included in the research coordinator log.

### 2.3. Data Analysis

Quantitative assessment of participation in the program includes descriptive information about the frequencies and response rates. Response rates were compared across demographic categories using chi-square tests. Qualitative data from participants were audio-recorded and transcribed. A summary was created immediately following the interview to capture key points and assist with codebook development. A list of codes was developed by the study team based on the semi-structured interview guide. Two members of the study team independently coded each interview and disagreement in assignment or description of codes was resolved through discussion between investigators or through modifying code definition. Quantitative and qualitative results were synthesized using a team process within and across sites.

## 3. Results

**Figure 1:**
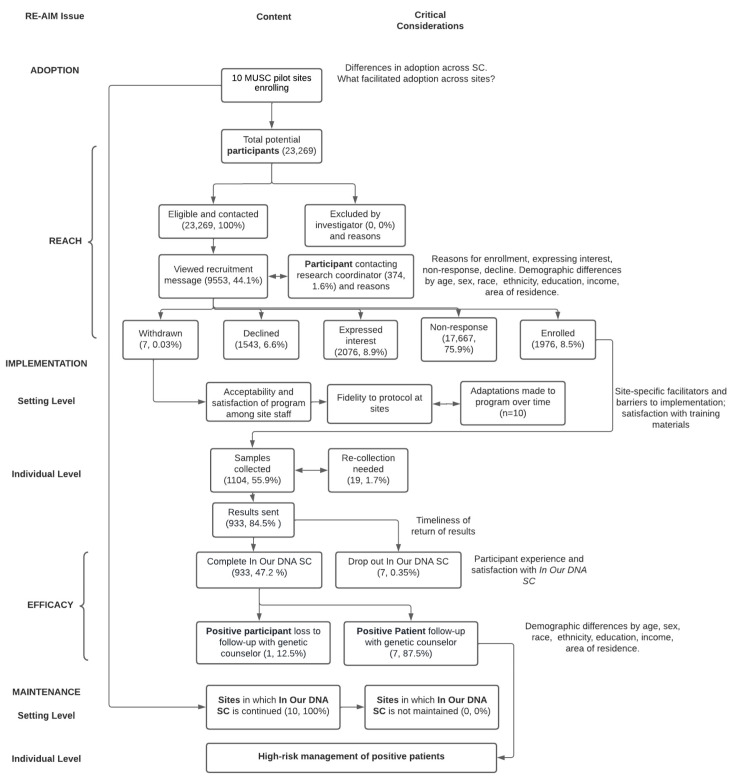
Consort Diagram. Adapted from Glasgow and Chambers (2012). Developing Robust, Sustainable, Implementation Systems using Rigorous, Rapid, and Relevant Science.

Between 8 November 2021, and 7 March 2022, 23,269 patients were approached through the patient portal (Epic’s MyChart) for recruitment across 10 clinical sites. Participants were followed through 20 July 2022. Most sites (80%) were located in Charleston County, with two sites affiliated with the MUSC regional health network outside of Charleston. A total of 2 of the 10 sites were OB/GYN practices, and 5 sites were family medicine/primary care practices (Figure 1).

The characteristics of individuals who participated in qualitative interviews (*n* = 20) included 15 White individuals (75%), 5 African American individuals (25%), a median age of 56.5 years, and a proportion of 40% male participants (*n* = 8). Participation in the *In Our DNA SC* program included 45% who enrolled in the program (*n* = 9), 45% who were non-respondents/undecided about enrollment (*n* = 9), and 10% who declined to enroll (*n* = 2).

### 3.1. Assessing Reach of In Our DNA SC

A total of 23,269 patients were contacted about the *In Our DNA SC* program, as of 20 July 2022, 1976 had enrolled (8.5% enrollment rate). Those who enrolled were predominately female (74.65%), White (84.51%), and had a median age of 50.1 years (Table 1). A total of 211 unique zip codes were represented among enrolled participants in South Carolina.

Table 2 provides detailed information about response rates with respect to enrollment, interest, decline, non-response, viewing of study information, and sample collection. The enrollment rate was higher in women (8.91%) than men (7.45%) (*p* = 0.0003) and was more than three times higher among White (10.5%) and Asian (8.71%) populations than among Black individuals (3.46%) (*p* < 0.0001). The rate of decline was similar in men and women (*p* = 0.2776), but higher among White individuals (7.29%) than Black (5.22%) or Asian individuals (5.57%) (*p* < 0.0001). Females were more likely to view the initial invitation (42.7%) than males (37.0%) (*p* < 0.0001). Black individuals were least likely (28.9%), while White participants (46.2%) were most likely to view the program invitation (*p* < 0.0001). Individuals between 30–39 years were most likely to view the initial invitation (44.4%). Enrollment was highest among individuals between 40–49 years old (10.3%).

Qualitative findings further illuminate the limitations of using the MyChart patient portal as a recruitment strategy, as many individuals were not aware of the initial invitation or did not recognize the invitation when asked. For example, one non-responder indicated, “Not that I could think of. I mean the biggest problem I don’t check… I don’t go into my chart that much you know unless my kids tell me I got a message or something and then I could think that’s how I saw it. I saw it when I first logged into it, it said I had a message” (55-year-old African American male, interested in study).

Other factors that influenced reach, or barriers to participation were primarily related to how data would be used. Specifically, many participants indicated concern about privacy and security of data and the impact participation would have on health or life insurance (Table 3). Those that were concerned about privacy and security cited worry about non-MUSC institutions gaining unauthorized access to their data, with one participant indicating, “I just don’t want my genetic information out there […] if someone were to hack into it then you know that could come back to you later on and say oh we know you’ve got this this and this and I’m just like I don’t I would not be comfortable with just having that out there” (68-year-old White male, declined to participate in study). Relatedly, participants were concerned about the impact of DNA data on health or life insurance. One respondent highlighted, “Literally, the only reason I’m not participating, it’s because of the fact that it’s part of my medical record and I’m trying to get life insurance. That’s really the only reason” (40-year-old White female, interested in study). Some individuals did not wish to participate because they preferred not to know about their health information. For example, one stated, “Well, I think it would be very scary to find out the results. I mean if people are anxious and get the results, how will they? I guess what I would want to know is what do I do with the information and who can help me navigate that?” (68-year-old White female, interested in study).

Facilitators to participation that helped increase reach included a family history of a condition, prior involvement in genetic testing, self-interest, and altruism. Participants often reported a strong family history of diseases associated with the Tier 1 conditions being tested. For example, one participant stated, “because I am a breast cancer survivor and my mother died of breast cancer and my father died of colon cancer, so those things are near and dear to me” (68-year-old White female, interested in study). Other participants highlighted that a lack of knowledge about medical history triggered their interest in testing, as one participant indicated, “Well the first reason is I don’t know a lot of my family history […] I didn’t know a lot of my grandparents […] some of the things that I go through may be because it’s just a genetic thing” (60-year-old African American female, interested in study). Prior involvement in clinical genetic testing or personal history of cancer was also a motivating factor for participation. For example, one participant indicated, “I am a cancer survivor, so those things kind of were the benchmarks for me” (34-year-old White female, interested in study). Finally, participants cited joining the study to support the greater good and future research findings. For example, one stated, “The more they learn, the more accurate they can get. The more you get to know” (40-year-old White female, interested in study) and recognition that this approach is the future of medicine, “It seemed to me like that’s kind of the future of where this thing is going, where you can actually use someone’s DNA to maybe give them a chance at knowing what their future could be” (47-year-old White male, enrolled in study).

### 3.2. Implementation of In Our DNA SC

Implementation of the population screening program was assessed at both the setting level and the individual level. A detailed assessment of setting-level implementation is underway to consider the acceptability and satisfaction of the program among clinical staff who were responsible for sample collection, fidelity to the protocols, and adaptations made to *In Our DNA SC* over the pilot period. We report on individual level implementation constructs.

A total of 1104 samples (55.9%) have been collected so far from those enrolled in the pilot phase of the program. The sample collection rate overall for those invited was 4.74%. Those who provided samples were predominantly female (72.83%) and White (86.96%) with a median age of 50.3 years (Table 4). A total of 19 (1.7%) of initial samples required recollection, which occurred through shipment of a sample collection kit directly to individual’s homes.

Qualitative findings provide detail about potential barriers and facilitators to sample collection. While there were not significant differences in collection by demographic groups, over half of those enrolled ultimately had their sample collected (58.5%). Barriers to sample collection primarily included modifications made to the appointment at which the sample was going to be collected and the distance required to travel to provide the sample. For example, research coordinator tracking logs included questions about how to provide a sample after a missed appointment or failure to collect, “My provider had to cancel my scheduled appointment tomorrow morning […] is there a way to reschedule when I provide the sample for the research study?” Distance was also a common concern, “I would have to drive to Charleston depending on what day it was […] it just depends on whether it was worth it for me” (49-year-old African American female, declined to participate study).

Implementation facilitators for in-clinic collection included efficient collection processes and enthusiastic staff. For example, “They did all my normal office visit stuff […] walked into the room and it’s like boom boom boom you know very quick getting all the free stuff done before the doctor came in […] while you are waiting for her go ahead and spit in the tube and here’s a pamphlet if you need more information” (60-year-old White female, enrolled in study).

### 3.3. Effectiveness of In Our DNA SC

The effectiveness of *In Our DNA SC* was assessed through assessing the proportion of individuals who completed the program (*n* = 1104, 58.5%) compared to those who dropped out (*n* = 7, 0.35%). In total, 8 of those with samples collected (0.72%) were found to be positive for a Tier 1 condition and 7 (87.5%) followed-up with a genetic counselor. One of the participants who declined genetic counseling was already aware of their positive result and the other was unable to be reached. All individuals were able to schedule their genetic counseling appointment within one week of results disclosure.

Our qualitative assessment of participant experience and effectiveness of aspects of the program included a need to simplify and shorten the initial consent form to help ensure people understand what they are committing to as part of the program. Participants recommended the study team modify the consent form to ensure it is “more digestible” and “not as intimidating” (58-year-old White female, interested in study) by offering “synopsis of what it was and answer questions that I may have” (59-year-old White female, interested in study). Opportunities to promote better understanding of the program included sharing what will be sent to the participants after they enroll (43-year-old White female, enrolled in study) and aligning the next steps with what it means for participants, “So I did all of this and it is helpful research to the state of South Carolina. But what does this mean for me? How do I navigate it and am I going to do anything with it? I mean, that’s a personal choice, but how do I get that information?” (68-year-old White female, interested in study).

## 4. Discussion

The goals of *In Our DNA SC* include population-level screening for actionable Tier 1 genetic conditions and fostering ongoing translational genomics research. Identifying an individual’s risk can allow for proactive screening for treatable conditions, which can enable precision-based clinical engagement of subpopulations who could benefit most. During the pilot phase of the program, we assessed program reach, implementation, and effectiveness.

We found low overall engagement, or reach, among racial minority individuals throughout the pilot phase of the program. Although Black individuals comprised approximately 30% of those initially invited, they were significantly less likely to open the initial MyChart recruitment message and less likely to take further actions of declining or enrolling in the study. Lower participation of racial and ethnic minority populations has been well-documented in the literature, with most genome-wide association study participants (81%) being of European ancestry [10,11]. However, wide-scale participation in genetics-based research and services is critical to accurately represent genetic diversity from a broad range of populations to avoid genetic misdiagnosis in these communities, and to facilitate the development of effective prevention strategies and personalized therapies for individuals of all backgrounds [12,13]. Reasons for poor participation in genomic research among racial and ethnic minority groups are complex. Our findings further support the range of barriers for participation, including concerns about privacy and the disclosure of results [14,15,16], historical transgressions and mistrust [17], and being unaware of research opportunities [18,19].

Alternative models to facilitate recruitment and retention of diverse participants into population wide genomic screening are needed to avoid perpetuating existing disparities in genetic research and access to genetic services [20,21]. Notably, a growing body of research suggests that minority participation in genetic studies is not due to a lack of interest, but rather due to deployment of unsuccessful and inconsistent recruitment strategies that do not adequately address the engagement preferences of diverse populations [19,22,23,24,25,26]. Although patient portals are increasingly used for research recruitment, these approaches have been found to result in bias toward younger, White populations [27,28,29,30]. We observed similar findings as we deployed messaging through MyChart (Epic’s patient portal). Recruitment of minority participants may require more robust stakeholder engagement using high-touch, relationship-centered community outreach efforts where those who initially engage racial and ethnic minority participants often becoming additional points of contact for participants throughout the duration of the genetic services or research [31,32]. Further, messaging that describes the transparency of study procedures, clear descriptions of safeguards and participant privacy, and emphasizing community-based recruitment can support the engagement of racial and ethnic minorities in genomic research [32,33]. As *In Our DNA SC* expands, we have incorporated efforts to increase the reach and representativeness of our population. These include the development of a community advisory board with representation from organization across South Carolina, the adoption of a diversity, equity, and inclusion statement for the program, the expansion of recruitment strategies to include community events and at home collection, as well as high-touch outreach through text message follow-ups and phone calls to individuals who express interest. Other opportunities include community capacity building or equipping already established, trusted groups, such as community health workers, to understand and participate as partners in genomic research [34,35,36].

Our focus on recruiting from and then collecting samples through clinical sites may have also contributed to the observed lower view rate of invitations among men (37.0%) compared to women (42.7%) and overall lower enrollment of males (7.45%) compared to females (8.91%). In addition to sites being skewed toward female populations (2 of 10 sites were OBGYN specialty sites), 5 sites were family medicine/primary care practices. Female gender is associated with portal-based communication [37] and the likelihood of engaging in clinical encounters. Interestingly, prior research has found that men are more likely to have higher trust and be willing to donate DNA and health data compared to females [38,39]. Thus, overrepresentation of females in our population may be due to our focus on recruitment primarily through clinical encounters and patient portals, as opposed to concern about the type of research being conducted.

The initial rate of sample collection (55.9%) is on par with collection rates among other population genomic screening programs [7]. Notably, DNA sample collection during the pilot phase occurred only in clinical sites and faced challenges with clinical encounters during the COVID-19 Omicron surge. During the Omicron surge, there was an increased number of cancelations, telehealth visits, and rotating staff in the clinical sites. Research coordinator tracking logs describe a participant reaching out to request another appointment to ensure their sample is collected. While each clinical site identified a provider champion and site administrative lead, all recruitment occurred outside of the clinical setting (e.g., providers and staff were not responsible for consenting). Previous reviews have emphasized the value of provider champions and primary care providers for enrollment [6]. Since the pilot phase, we have further expanded clinical sites that are collecting samples and implemented enhancements to training for provider champions and clinical site leads to increase engagement and understanding of *In Our DNA SC*. We have also implemented other sample collection opportunities, including through drop-off at events facilitated by research staff and through at-home mail kits.

Finally, our assessment of the effectiveness of the *In Our DNA SC* program provided information about how well we achieved the primary public health goal of identifying individuals with Tier 1 conditions of hereditary breast and ovarian cancer, Lynch syndrome, and familial hypercholesterolemia. Among those who provided a sample during the pilot phase of the program, we found 8 individuals (0.72%) who were positive for Tier 1 conditions. This screen positivity rate aligns with screening positivity rate for other programs screening for Tier 1 conditions of approximately 1-2% of the population [40]. Of those who screened positive for a condition, all but two completed follow-up genetic counseling and were scheduled within one week of results disclosure. This model of an individual presenting for genetic counseling due to a positive genetic test result shifts the paradigm away from the traditional pre-test, post-test counseling model. A 2017 survey of genetic counselors indicated support for population-based genetic screening within the next 10 years. Recommendations to support these growing population screening initiatives include the education of non-genetic providers, deployment of genomic application toolkits for local clinics in preparation for population-based screening, and adoption of new service delivery models to address concerns about pre-test counseling and informed consent and the collection of personal and family medical history to inform clinical management and cascade testing.

Our approach is not without limitations. As part of the pilot of the population screening program, we focused only on recruitment in clinical settings, limiting our overall reach to participants. While this was a necessary step to support the technical aspects of implementation, other forms of recruitment and outreach will be critical to ensure representativeness of the South Carolina population. Additionally, the assessment of our program primarily focuses on individual-level barriers and facilitators to reach, implementation, and effectiveness. Factors beyond the individual level were described as part of qualitative interviews (e.g., concern about insurance policies) and noted during sample collection (e.g., clinical workflow and site-specific barriers to sample collection); however, we did not focus on these as part of the present evaluation. Further, our assessment of the program did not explicitly include “adoption” and “maintenance” of the RE-AIM framework. Adoption was not included in this evaluation, given that the program was an institutional priority, all clinical sites were required to adopt the program. The assessment of maintenance at the clinic or site level (e.g., continued use of *In Our DNA SC* workflow) and individual level (e.g., high-risk management) is currently outside of the scope of our early findings but is planned to be included as part of the ongoing evaluation of the program.

Population screening offers a unique opportunity to integrate precision-based approaches across clinical and public health settings. As access to population-based screening grows, it is critical to identify outcomes and develop strategies to rapidly assess progress toward these goals. The use of implementation science can help better understand how to support the success of *In Our DNA SC* and ensure the sustainability of population-level genetic testing. Ultimately, this approach supports MUSC’s efforts to use learning health system strategies where implementation research questions are evaluated at the point of care. Such approaches will eventually allow us to realize the promise of population genomics screening and maximize the utility of precision-approaches for individuals in our community and multidisciplinary teams of researchers and providers. The model-based components of our evaluation program can help support the generalization of lessons learned from *In Our DNA SC* and the identification of best practices to streamline the expansion of similar population genomics programs at other institutions.

## Figures and Tables

**Table 1:** Demographic characteristics of patients invited to participate, enrolled, and who provided samples for *In Our DNA SC* during the pilot phase.

	Total *n* (%) (*n* = 23,269)	Enrolled *n* (%) (*n* = 1976)	Sample *n* (%) (*n* = 1104)
**Gender**			
Female	16,548 (71.12)	1475 (74.65)	804 (72.83)
Male	6721 (28.88)	501 (25.35)	300 (27.17)
**Race**			
Black	6454 (27.74)	223 (11.29)	102 (9.24)
White	15,880 (68.25)	1670 (84.51)	960 (86.96)
Asian	287 (1.23)	25 (1.27)	15 (1.36)
Other	547 (2.35)	54 (2.68)	25 (2.26)
Missing	101 (0.43)	5 (0.25)	2 (0.18)
**Ethnicity**			
Hispanic/Latino	454 (1.95)	53 (2.68)	19 (1.72)
non-Hispanic/Lat	22,477 (96.60)	1902 (96.26)	1078 (97.64)
Missing	338 (1.45)	21 (1.06)	7 (0.63)
**Age**			
18–29 years	3631 (15.60)	250 (12.65)	111 (10.05)
30–39 years	4111 (17.67)	403 (20.39)	213 (19.29)
40–49 years	3217 (13.83)	332 (16.80)	174 (15.76)
50–59 years	3411 (14.66)	320 (16.19)	192 (17.39)
60–69 years	4056 (17.43)	333 (16.85)	193 (17.48)
70–79 years	3522 (15.14)	284 (14.37)	188 (17.03)
80–89 years	1153 (4.96)	50 (2.53)	30 (2.72)
90+ years	168 (0.72)	4 (0.20)	3 (0.27)

**Table 2:** Response rates for the enrolled, interested, declined, identified, viewed invitation, and sample collected groups out of all patients invited to participate in the total population and stratified by demographic categories.

		Mutually Exclusive Categories out of All Patients Sent Recruitment Messages ^1^								Viewed Invitation ^2^ (*n* = 9553)		Sample Collected ^3^ (*n* = 1104)	
		Enrolled (*n* = 1976)		Interested (*n* = 2076)		Declined (*n* = 1543)		Non-Response (*n* = 17,667)					
	*n*	%	*p*-Value	%	*p*-Value	%	*p*-Value	%	*p*-Value	%	*p*-Value	%	*p*-Value
**Total**	**23,269**	**8.49**	**-**	**8.92**	**-**	**6.63**	**-**	**75.9**		**41.1**		**4.74**	
**Gender**			**0.0003**		**<0.0001**		**0.2776**		**<0.0001**		**<0.0001**		**0.1990**
Female	16,548	8.91		9.55		6.74		74.8		42.7		4.86	
Male	6721	7.45		7.38		6.35		78.8		37.0		4.46	
**Race**			**<0.0001**		**<0.0001**		**<0.0001**		**<0.0001**		**<0.0001**		**<0.0001**
Black	6454	3.46		6.21		5.22		85.1		28.9		1.58	
White	15,880	10.5		10.0		7.29		72.1		46.2		6.05	
Asian	287	8.71		8.36		5.57		77.4		42.5		5.23	
Other	547	9.69		8.41		5.12		76.8		34.9		4.57	
Missing	101	4.95		13.9		4.95		76.2		43.6		1.98	
**Ethnicity**			**0.0166**		**0.2135**		**0.0695**		**0.1270**		**0.5471**		**0.0552**
Hispanic/Latino	454	11.7		11.0		3.96		73.4		40.8		4.19	
non-Hispanic/Lat	22,477	8.46		8.90		6.69		75.9		41.1		4.80	
Missing	338	6.21		7.69		6.51		79.6		38.2		2.07	
**Age**			**<0.0001**		**<0.0001**		**0.0018**		**<0.0001**		**<0.0001**		**<0.0001**
18–29 years	3631	6.89		7.22		5.23		80.7		38.2		3.06	
30–39 years	4111	9.80		8.29		7.15		74.8		44.4		5.18	
40–49 years	3217	10.3		10.2		6.12		73.3		43.5		5.41	
50–59 years	3411	9.38		10.1		6.45		74.1		41.7		5.63	
60–69 years	4056	8.21		9.81		6.95		74.9		42.2		4.76	
70–79 years	3522	8.06		9.48		7.58		74.8		40.5		5.34	
80–89 years	1153	4.34		5.55		6.68		83.4		29.2		2.60	
90+ years	168	2.38		2.98		9.52		85.1		24.4		1.79	

^1^ In total, 7 of the 23,269 people withdrew from the study and are not in the mutually exclusive categories; ^2^ Viewed invitation is not mutually exclusive, patients who viewed study invitation entered into the enrolled, interested, declined, or identified categories, but patients did not need to view the invitation to enter into these categories; ^3^ Sample collected is a subset of those enrolled. *p*-values are chi-square results comparing the column percent (i.e., enrolled, interested, declined, non-response) by row categories (i.e., Are the percent enrolled the same in males and females?). Only 1 patient who declined did not view the invitation; 124 who enrolled did not view the message.

**Table 3:** Qualitative Themes and Quotes.

Theme	Quotes	Demographics
**REACH: Barriers to Participation and Resulting in Reduced Reach**		
Concern about privacy and security of data	I mean is it just you know I just… With all the DNA stuff going on now I just kind of wonder	African American, 55 years old, male, interested
	The security of any specific findings associated with my medical I don’t know individual medical circumstances. What entities would have access to it and when and what purposes and this kind of thing	White, 53 years old, male, interested
	I just don’t want my genetic information out there […] I think you probably have a wonderful computer program within your institution but if somebody was to hack into it then you know that could come back to you later on and say oh we know you’ve got this this and this and I’m just like I don’t I would not be comfortable with just having that out there	White, 68 years old, male, declined
Impact of participation on health or life insurance	Well, I wouldn’t be real thrilled with insurance companies having free range or goes through my medical records but I mean the opportunity for studies and for additional research I don’t care who makes the buck off of it as long as it’s helpful.	White, 53 years old, male, interested
	Well like I said the questions I had about whether the insurance companies get access to this information particularly since it’s a state function that would concern me that my insurance rates might be adjusted up because of a marker that they found in the DNA that was my concern when I you know I sat there and thought about it for a few minutes first as I don’t know okay but I said you know it’s feasible that might happen Don’t know that it would but do I really want to go through all that so I said probably not and that’s what I had declined.	White, 68 years old, male, declined
	No, I mean I’m interested in it. Literally. The only reason I’m not participating, it’s because of the fact that it’s part of my medical record and I’m trying to get life insurance. That’s really the only reason.	White, 40 years old, female, interested
	Who will have access to my information? Specifically, I wouldn’t want an insurance company to have access and deny me insurance because I may be a high-risk person to insure if something shows in my test.	Research coordinator tracking log
	I wanted to participate in this study, but the risks are too high. I can’t afford to pay a higher health insurance premium. I live on social security. If you can’t protect my data so it can’t be traced back to me by an insurance company, I have to refuse.	Research coordinator tracking log
Do not want to know results	Yeah, very well surprising but again my dad had it two or three of his brothers and sisters in both of his grandparents so you know that’s a real touchy subject with me so that’s another part of the reason I don’t want that information going out there. I’ve never had a test to see if I have the gene or whatever it is and to be honest with you at my age, I don’t know I particularly want to know that information that’s probably a worst-case scenario for any health issue that I have and I really don’t want to. You know, I think I would be more depressed by finding out the results than anything else and I don’t really need that kind of negativity in my life I’ve already has no health problems as it were.	White, 68 years old, male, declined
	Well, I think it would be scary to find out the results. I mean if people are anxious and get the results, how will they? I guess what I would want to know is what do I do with this information and who can help me navigate that?	White, 68 years old, female, interested
Unaware of consent	That’s a hard one, because my chart and getting those messages is actually really effective. I really don’t remember seeing a message about any of the consent form in my regular email, which I do normally get for and I get noticed notifications in my chart.	White, 34 years old, female, interested
	Well actually I am one of those that kind of just glances over things and so it probably was there the whole time.	White, 43 years old, female, enrolled
	Not that I could think of. I mean the biggest problem I don’t check… I don’t go into my chart that much you know unless my kids tells me I got a message or something and then I could think that’s how I saw it. I saw it when I first logged into it, it said I had a message.	African American, 55 years old, male, interested
**REACH: Facilitators to Participation Resulting in Increased Reach**		
Family history of a condition	Yes, because of a couple things that they’re that you’re looking at. And because I am a breast cancer survivor and my mother was died of breast cancer and my father died of colon cancer, so those things are near and dear to me. And if there’s something that I need to know or need to tell my biological son, you know that I think it’s important that he has information.	White, 68 years old, female, interested
	Well, we have in my family there’s colon cancer is a big thing there’s been five of my family members that have had it I’ve not. I get tested every five years of all my doctors are at MUSC.	White, 68 years old, male, declined
	The first reason is I don’t know a lot of my family history. I mean I know my mom, my dad, but I didn’t know a lot of my Grandparents so Some of my medical history. Some of the things that I go through might be because it’s just a genetic thing, you know.	African American, 60 years old, female, interested
Involvement in prior genetic testing or personal history	I don’t know what’s too much to share in this, so apologies if I ramble. But I suppose I have a lot of you know, history with cancer and my family, and I think that was kind of one of the big things that you know. I’m also a cancer survivor, so those things kind of were the benchmarks for me. So, if something came up came up that was related to that. For example, I would probably be more moderately interested then genetics, but I’m also still interested in this. You know, until something else were to arise.	White, 34 years old, female, interested
	I don’t recall having any questions about it or concerns because of the fact that I have already done DNA testing previously, so it didn’t. It didn’t bother me. I mean, maybe some other people might be concerned about privacy.	White, 60 years old, female, enrolled
Interested in results for self	I think for personal reasons I’d like to know if there’s like a potential issue that I could avert.	White, 40 years old, female, interested
For the greater good	Well, you’re DNA is already out there. Whether you wanted to or not, I opted to share it because I wanted it. The more they learn, the more accurate they can get. The more that you get to know.	White, 33 years old, female, enrolled
	And like I said it, if it can help somebody else, maybe down the road, then I think that’s a good thing.	African American, 60 years old, female, interested
	No, you had mentioned about different research opportunities. I just click yes, that I’d be interested in entertaining things that come through my chart. Um, I’m not going out and looking to make a living on doing research studies like you know, I, but to the extent that it’s, you know, a couple times or something like that because I’m so close to the university, I do feel like, you know, it’s easy enough to be helpful.	White, 58 years old, female, interested
	And it, and it seemed it seemed to me like that’s kind of the future of where this kind of thing is going, where you can actually use someone’s DNA to maybe give them a chance at knowing what their future could be.	White, 47 years old, male, enrolled
**IMPLEMENTATION: Barriers to Adherence to Protocols/Fidelity to Protocol at Individual or Clinical Level**		
Modifications made to appointment associated with sample collection	It could happen whenever I go into the clinic. That’s what I thought, but I don’t go into. I usually do the virtual visits if I can, unless it’s for some reason I need to be face to face with my doctor.	African American, 29 years old, female, enrolled
	What can I do to get tested now that the clinic failed to test me?	N/A
	Hey there, my provider had to cancel my scheduled appointment tomorrow morning. I will be rescheduling with her at a later date. Is there a way to reschedule when I provide the sample for the research study?	N/A
Distance to provide sample	If possible, it just depends on what it would be worth my while because I would have to drive to Charleston depending on what day it was. And you know if I could drive from work, work is closer to Charleston but still 45 min there and back […] so it just depends on whether it was worth it for me.	African American, 49 years old, female, declined
	Does participating in this survey require regular trips to MUSC? I live about 2 h away and regular trips are needed; I cannot participate.	N/A
	Is there anywhere in the upstate where this saliva submission can be done? Driving to Charleston is about 3 h. Also, from the list of locations provided, would need to know which is the most “northern” so I don’t have so far to drive if I have to.	N/A
**IMPLEMENTATION: Facilitators to Adherence to Protocols/Fidelity to Protocol at Individual or Clinical Level**		
Efficient collection process	Yeah, I was provided a plastic, I guess vial with the top and it was explained that I need to get my saliva to a certain line and then close up the vile and go back.	White, 34 years old, female, interested
	They did all my normal office visit stuff. You know, blood pressure or temperature. All that kind of stuff. And wow, ‘cause I actually had two people in the room so one was doing the blood pressure and everything and typing stuff in the chart. The other one said okay, we understand, you know was there and said we have this too. When you’re done filling out all your paperwork. ‘cause I mean my visit was fast. I got there for my appointment. Barely sat down less than five minutes. I was called in the back to get. Now my other stuff walked in the room and it’s like boom boom boom boom you know very quick getting all the free stuff done before the doctor came in and then it’s like okay um the doctor will be in in just a second while you’re waiting for her go ahead and spit in the tube and here’s a pamphlet. Two if you need further information so.	White, 60 years old, female, enrolled
Staff enthusiasm and relationships	No, I will give you a comment on my doctor’s office. In particularly the nurse that was taking my information and getting the saliva sample seemed very excited about it and actually commented that it was a great thing to do, and she was glad to see people do it.	White, 47 years old, male, enrolled
**EFFECTIVENESS: Facilitators to Effectiveness of Completing Public Health Goal of Identifying** **High-risk Individuals**		
Need to simplify and shorten consent	So, it’s okay, but it’s kind of like if somebody sat me down and you know gave me a synopsis of what it was and answered any questions that I may have and discussed What’s the downside might be versus the upsides.	White, 59 years old, female, interested
	No, I think it was I mean it was several pages long the whole thing but I think it was fairly straight forward that my only questions had to do with security or I guess it’s best said security of the medical of any kind of findings you know where they’d be released where they wouldn’t be and it appears it’s going to be pretty secure so I was comfortable signing it.	White, 53 years old, male, interested
	Uhm, maybe a little more explanation. I, I know legally you have to put a lot of information about what could possibly happen if someone gets your DNA results or in the future, which is kind of scary. So maybe just a little verbiage to kind of make people feel better. That may be on the fence.	White, 47 years old, male, enrolled
	And because of that, yes, that’s good. It takes 20 min to go through. I just think you’re going to lose a lot of people because people don’t have the attention span. Understand what it means. It’s intimidating and just you’re going to lose people. But if you say, here’s the gist of it and throughout it have hyperlinks to click on it, then it’s more digestible. Or not as intimidating.	White, 58 years old, female, interested
Better understanding of program	I guess how quickly I’m going to get results. What kind of stuff you are actually going to be sending me with my DNA and then what you all are using our DNA to study like why you’re collecting it?	White, 43 years old, female, enrolled
	I really value kind of the full story, almost I would almost be more inclined to have the information up front at the beginning to say hey. You know this is this is the purpose and like why this is important, and then at the end if it was possible to kind of have that information to say this is kind of what you’re participation led us to find and what we were able to discover in general with the program. Like you know, kind of a findings summary for me, so it’s sort of like that. I don’t need to necessarily know all the method and what happened like how it was conducted in the process. I think just more of the purpose and then the results are more what I’m most would be most interested in.	White, 34 years old, female, interested
	Yes, because okay. So, I did all this, and I did this helpful research to the state of South Carolina. But what does this mean for me? How do I navigate it and am I going to do anything with it? I mean, that’s a personal choice, but how do I get that information?	White, 68 years old, female, interested

**Table 4:** Sociodemographic Characteristics of Samples Collected.

	Enrolled	Sample Collected	
	*n*	Percent (*n*)	*p*-Value
**Total**	**1976**	**55.9 (1104)**	**----**
**Gender**			**0.0364**
Female	1475	54.5 (804)	
Male	501	59.9 (300)	
**Race**			**0.0096**
Black	223	45.7 (102)	
White	1670	57.5 (960)	
Asian	25	60.0 (15)	
Other	53	47.2 (25)	
Missing	5	40.0 (2)	
**Ethnicity**			**0.0012**
Hispanic/Latino	53	35.9 (19)	
non-Hispanic/Lat	1902	56.7 (1078)	
Missing	21	33.3 (7)	
**Age**			**<0.0001**
18–29 years	250	44.4 (111)	
30–39 years	403	52.9 (213)	
40–49 years	332	52.4 (174)	
50–59 years	320	60.0 (192)	
60–69 years	333	58.0 (193)	
70–79 years	284	66.2 (188)	
80–89 years	50	60.0 (30)	
90+ years	4	75.0 (3)	

## Data Availability

Data are available upon request to the corresponding author.
